# Asexual Recombinants of *Plasmopara halstedii* Pathotypes from Dual Infection of Sunflower

**DOI:** 10.1371/journal.pone.0167015

**Published:** 2016-12-01

**Authors:** Otmar Spring, Reinhard Zipper

**Affiliations:** Institute of Botany, University of Hohenheim, Stuttgart, Germany; Agriculture and Agri-Food Canada, CANADA

## Abstract

Genetically homogenous strains of *Plasmopara halstedii* differing in host specificity and fungicide tolerance were used to test the hypothesis that asexual genetic recombination occurs and may account for the high genotype diversity of this homothallic reproducing oomycete, which causes downy mildew in sunflower. Dual inoculation of sunflower seedlings with single zoospore strains of complementary infection characteristics caused sporulation under conditions where inoculation with each strain alone failed to infect. PCR-based investigation with strain-specific primers proved the presence of genetic traits from both progenitors in single sporangia collected from sporangiophores of such infections. Sister zoospores released from these sporangia revealed the genotype of the one or the other parental strain thus indicating heterokaryology of sporangia. Moreover, some zoospores showed amplification products of both parents, which suggests that the generally mononucleic spores derived from genetic recombination. The possibility of parasexual genetic exchange in the host-independent stage of infection and the evolutionary consequences are discussed.

## Introduction

Genetic variation in populations of plant pathogenic oomycetes is fundamental for the evolutionary potential, particularly when the host spectrum is limited or breeding for resistance in crop plants narrows the host accessibility and requires permanent adaptation. Sexual recombination and interspecific hybridization are predestined events to increase the genetic variation, but many oomycetes reproduce through homothallic gametangiogamy or apomixis [1] and natural hybridization has rarely been proven in oomycetes [2], except for some hemibiotrophs such as *Phytophthora* [3–5] and *Pythium* species [6]. Parasexual events have been suggested as an alternative explanation for the unexpected high phenotypic diversity in some plant pathogenic oomycetes [7–8]. In *Bremia lactucae*, such events were seen as a factor responsible for the hyperploidy or heterokaryon situation found in some Australian and Californian isolates [9].

**Fig 1:**
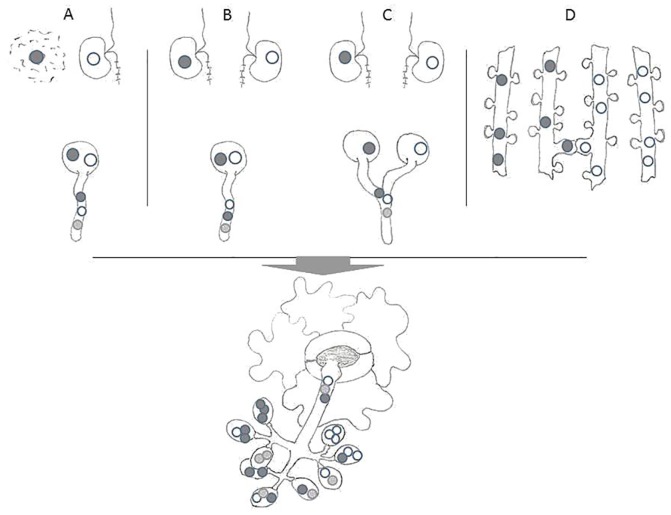
Theoretical modes of parasexual genetic exchange in *Plasmopara halstedii* within host-independent (A-C) and host-dependent (D) stages of infection. A, uptake of nucleus from bursted zoospore of strain I into zoospore of strain II (as proposed by Gu and Ko [16]); B, zoospore fusion (as suggested by Ersek et al. [15]); C, fusion of germtubes from sporocysts; D, hyphal anastomosis (as proposed by Hammer and Spring [17]). Heterokaryon situation (black and white nuclei) may finally lead to recombination (grey nuclei) and result in sporangia with uniform parental or mixed genotype as well as monokaryotic zoospores of either strain I or strain II or a new recombinant type.

A conspicuous high phenotypic plasticity has been reported for *Plasmopara halstedii*, the causal agent of downy mildew in sunflower [10–12], although sexual reproduction of single zoospore isolates has shown its homothallic nature, a feature that usually constrains genetic diversity [13]. Previously, dual inoculation of sunflower under selection conditions (different host resistance and fungicide tolerance) with two phenotypically different single zoospore strains of the downy mildew pathogen resulted in infections that normally could not be overcome by either of the strains alone. The mitotically produced sporangia of such infections gave rise to a new, recombinant phenotype with characteristic DNA traits in the F1 and following sporangial generations, albeit sexual recombination of the parental strains had been excluded by the experimental design [14]. This experiment provided evidence for parasexual gene transfer in *P*. *halstedii*. However, it remained unclear how and when the genetic exchange took place. Observations of interspecific zoospore fusion [15] or uptake of nuclei in zoospores of *Phytophthora* [16] lent support to the possibility of a pre-infectional genetic exchange (Fig 1). Alternatively, fusion of germtubes from encysted zoospores before host penetration could be another way to form heterokaryotic hyphae, which might overcome the selective conditions that exclude infection of each of the parental strains alone. Finally, in *P*. *halstedii* exchange of nuclei and mitochondria between hyphae can occur via anastomosis [17]. Thereby providing a third possibility for somatic gene exchange which might take place under nonselective infection conditions or in an early stage of host invasion before resistance measures or fungicide activity could stop the pathogens.

In the present study, we report on new, recombinant-selective inoculation experiments that were intended to shed more light in the instant of time and mode of genetic exchange between different strains of *P*. *halstedii*. Moreover, we present a PCR-based assay for testing the genetic constitution of single sporangiophores, sporangia and zoospores from successful recombinant infections with respect to their potentially recombinant or heterokaryotic nature.

## Materials and Methods

### *Plasmopara halstedii* isolates and infection tests

Isolates of *P*. *halstedii* (Farl.) Berl. & De Toni used in this study were propagated on susceptible sunflower seedlings (*Helianthus annuus* L. cv. Giganteus) using the whole seedling inoculation (WSI) technique as described previously [18]. According to physiological race testings [19] on sunflower differential lines [20] and leaf disc assays for metalaxyl sensitivity [21], isolates differing in at least two phenotypic characters suitable for selective cultivation were identified and used for generating single zoospore strains in order to ascertain genetic homogeneity of the inoculum [22]. From this procedure, the following three strains resulted: Strain A, Ph1-00/IN-B5z, pathotype 703, sensitive to metalaxyl M at concentrations above 0.1 ppm; Strain B, Ph8-99/BL-A4z, pathotype 730, tolerant to metalaxyl at concentrations of 10 ppm and more; Strain C, Ph5-05/JU-B18z, pathotype 730, tolerant to metalaxyl at concentrations of 10 ppm and more. The infectivity of the strains on selected sunflower genotypes and the sensitivity for the fungicide metalaxyl M is shown in Table 1. Zoosporangia of the single spore lines used in this study are deposited in the collection of O. Spring, stored in the herbarium HOH of the Institute of Botany, University of Hohenheim, under the specimen numbers HOH-016622 (Ph1-00/IN-B5z), HOH-016619 (Ph8-99/BL-A4z), and HOH-016524 (Ph5-05/JU-B18z).

**Table 1:** Pathogenicity of parental strains. Infection behaviour of the *Plasmopara halstedii* strains **A**-**C** on sunflower differential lines and sensitivity (**s**) or tolerance (**t**) towards the fungicide metalaxyl M (1 ppm).

strain	control cv. Giga	PM 13	HAR 4	QHP 1	Metalaxyl tolerance
**A** IN-B5z	+	-	**+**	**+**	**s**
**B** BL-A4z	+	**+**	**-**	**-**	**t**
**C** JU-B18z	+	**+**	**-**	**-**	**t**

+ = infective;

- = not infective

### Dual inoculation experiments

Testings for possible phenotypic recombination were carried out using fresh sporangia (7500 / mL) of the strains A to C individually or in a 1:1 mixtures of A/B and A/C (Table 2) for whole seedling inoculation with pre-germinated (48h, 25°C on wet filter paper) dehulled sunflower germlings. Seedlings of the host genotypes cv. Giganteus (general susceptible), PM13 (resistant to strain A, susceptible for strain B and C) and QHP1 (susceptible for strain A, resistant to strain B and C) were used to ascertain the vitality and virulence behaviour of the used sporangia. In tests with mixtures of sporangia, QHP1 served as first selection marker to exclude infection with the less virulent strains B or C. Metalaxyl M (1 ppm final concentration) was added to the inoculation medium when zoospore release had started (ca. 2–3 h after watering the sporangia) and served as a second parameter for selecting the tolerant strain B and C and to inhibit infections by strain A. After 4h of inoculation in darkness at 16°C, seedlings were planted in sterilised soil and cultivated for 14–16 days in a climate chamber (18°C, 70% rel. humidity 14/10 h light/darkness) before sporulation was induced as described earlier [18]. Tests with low sporulation (less than 50% infected seedlings) in either of the individual control samples of the strains were discarded. Putative recombinants from dual inoculation experiments were recorded when sporulation has occurred on QHP1 in the presence of metalaxyl. Sporangiophores, sporangia and zoospores of such infections were used for subsequent analysis.

**Table 2:** Scheme for dual infection test and expected sporulation result in case of successful phenotypic recombination.

inoculum	H.a. cv Giganteus	host QHP1	QHP1 + 1 ppm metalaxyl
Strain A	+	+	-
Strain B	+	-	-
Strain C	+	-	-
Strain A+B	+	+	**+**
Strain A+C	+	+	**+**

+ = infective;

- = not infective

The expected infection behaviour of the used strains A-C according to their pathotype characteristics in single and dual inoculation experiments is listed in Table 2. Phenotypic recombination was defined when sporulation occurred on QHP1 in the presence of metalaxyl M (1 ppm). Sporangia of such infections were harvested and used for DNA extraction and microscopical studies.

### DNA extraction and primer development

Zoosporangia (1–3 mg) of the parental strains A-C were mechanically ruptured in a mixer mill (MM2, Retsch, Haan, Germany). DNA was isolated according to the protocol described previously [23]. The DNA pellet of the final step was resuspended in sterile deionised water (50 to 200μl depending on the initial amount of zoosporangia) and the concentration determined in a BioPhotometer (Eppendorf, Hamburg, Germany).

DNA samples (30ng) of strain A-C were used for PCR-amplification with 12 individual primers (S1 Table) in one-primer-PCR with RedTaq Mastermix 2x (Genaxxon Bioscience, Ulm, Germany), Taq (Fermentas, Thermo Scientific, Waltham, USA) and MgCl_2_ (50mM final conc.) at low annealing temperature under the following conditons: initial denaturation for 2 min at 94°C; 40 cycles with denaturation of 30 sec at 94°C; annealing of 30 sec at 36°C; elongation of 90 sec at 72°C; final elongation of 5 min at 72°C.

The amplification products of the PCR fingerprints were compared on agarose gels (1.5%) and polymorphic bands extracted (GeneJET Gel Extraction Kit Nr. K0691; Thermo Fisher, Waltham, MA, USA) for DNA sequencing. The seleceted amplicons were cloned (StrataClone PCR Cloning Kit Nr. 240205; Agilent, Santa Clara, CA, USA) and submitted to a commercial sequencer (Macrogene, Amsterdam, The Netherlands). Sequences were analysed using the basic local alignment search tool (BLAST, NCBI).

From PCR with the primer LR6-Om (5‘-CGCCAGACGAGCTTACC), two amplicons of 930 and 982 bp in size (S1 Fig arrow) were found to be suitable for differentiating among strain A and the two metalaxyl tolerant strains B and C. Sequence analysis revealed identity of the 982bp product for strain B and C. The 930 bp amplicon of strain A showed high similarity, but differed by a 52 nt deletion between positions 136 to188 (for sequences see supplementary material S2 Table). This difference was used to generate a pair of primers bridging the polymorphic part of the sequence. The primer pair INBL_WG_F1 (GAAGGGTCAGTGTCCACGCAAAT) and INBL_WG_R1 (GAACGTCGAAATTAACAGCCAAACATC) gave short PCR amplification products of 113 bp with DNA of strain A and 165 bp with strain B and C when applied at the following conditions: initial denaturation for 4 min at 94°C; 36 cycles with denaturation of 15 sec at 94°C; annealing of 15 sec at 65°C; elongation of 15 sec at 72°C; final elongation of 5 min at 72°C. PCR samples consisted of: 5 μL RedTaq mastermix 2x (Genaxxon Bioscience, Ulm, Germany); 5 μL H_2_O; 0.5 μL primer INBLWGF1 plus 0.5μL primer INBLWGR1, 0.5 μL template DNA (3 ng/μl). The PCR was performed in a Peqlab Master Cycler (Peqlab, Erlangen, Germany). The amplification products were analysed through microchip capillary electrophoresis (MultiNA, Shimadzu, Duisburg, Germany) according to the protocol of the manufacturer. Alternatively, agarose gel electrophoresis (1.5% and 2% agarose; SeaKem^®^ LE agarose, BMA, Vallensbaek Strand, Denmark) was applied and products stained with GelStar^®^ (BIOzym Diagnostik, Oldendorf, Germany) and photographed under UV illumination. E.A.S.Y Win 32 Version 2.02.127 software (Herolab, Wiesloch, Germany) was used for gel analysis. Sequencing of the 165 and 113 bp amplicons confirmed the expected sequences.

### Determination of PCR sensitivity

To determine the detection limits of the PCR-based sample identification, single sporangiophores, sporangia and zoospores of each of the strains were used. Sporangiophores were harvested individually with fine forceps from the surface of sunflower leaves under a dissection microscope (Leica S8 APO; Leica Microsystems, Wetzlar, Germany). Sporangia were spread on the surface of a microscope slide and collected individually with a fine nylon fiber fixed to a pin holder. Single zoospores were collected from 1% water agar plates after release from sporangia using the microcapillary technique [22]. This process was monitored under an inverse microscope (Hund, Wetzlar, Germany) which allowed to control that only single zoospores were sucked into the capilary (S2 Fig). In order to collect sister zoospores from single sporangia, the microcapillary was moved near the released zoospores which were trapped in the water film around the sporangium. By gently pressing the capillary on the agar surface, a trench was formed and filled with water so that the zoospores could move one by one into the channel-like depression. In this way they could be picked up one by one with the microcapillary.

Collected sporangiophores and sporangia were incubated in 5μL H_2_O for 3 h at room temperature and then frozen at -20°C until use. After thawing samples from sporangiophores/sporangia were treated in an ultrasonic bath for 30 sec and then directly used for PCR under the conditions described above. Samples from zoospores collected in water were dried at 45°C and afterwards dissolved directly in RedTaq master mix (11μL final volume). In case of single sporangia and zoospores, the number of cycles in PCR was raised to 38 and 40, respectively, in order to compensate for the low amount of template DNA in the samples. Detection of the strain genotype A-C was possible using single sporangiophores, single sporangia, and single zoospores (S3 Fig)

### Fluorescence microscopy

The karyological status of sporangia and zoospores was investigated with fluorescence microscopy (Zeiss Axioplan, filter combination UV 395–440 excitation/FT 460/ LP 470 barrier; documentation system Leica DMC2900 with software LAS V4.6) using 4′,6-diamidin-2-phenylindol (DAPI) or Hoechst 33342 as DNA staining dyes.

## Results

### Phenotypic recombinant infections from dual inoculation experiments

From 18 independent WSI experiments using 1:1 mixtures of strain A and B (11 experiments) and strain A and C (7 experiments) as inoculum, 14 resulted in infected QHP1 plants inoculated in the presence of 1 ppm metalaxyl M, whereas no infection occurred in 4 cases. The percentage of fungicide treated QHP1 plants showing sporulation on cotyledons two weeks post inoculation ranged between 30–50% and sporulation density was low in comparison to control infections on cv. Giganteus plants without metalaxyl.

### Molecular studies on phenotypic recombinants

Single sporangiophores, sporangia and zoospores of infected plants were used for the PCR-based determination of the pathogen genotype present in mitotically formed propagation structures. In all three cases, samples showing single amplicons of one of the parental genotypes as well as samples with both amplicons were found (Tables 3–6). When testing individual sporangiophores, samples showing only the amplicon of strain B and C made up 68% and 72%, respectively, whereas samples containing only the PCR product of strain A were rare (2%) or lacked completely (Tables 3 and 4). The ratio of samples showing both parental bands reached close to one-third (30% / 28%) in both tested combinations, A + B or A+C. This implies that sporangiophores of the phenotypic recombinant infections may derive from a heterokaryotic mycelium which produces sporangiophores with the genotype of either parent or of mixed type.

**Table 3:** PCR of sporangiophores. Amplification products detected in single sporangiophores collected from infected plants after dual inoculation with strain A+B (n = 4 inoculation experiments).

Test No.	No. of tested sporangiophores	PCR product ofType A	Type B	Type A+B
1	8	0	7	1
2	8	1	5	2
3	8	0	4	4
4	16	0	11	5
n = 3	40	1 (2%)	27 (68%)	12 (30%)

**Table 4:** PCR of sporangiophores. Amplification products detected in single sporangiophores collected from infected plants after dual inoculation with strain A+C (n = 3).

Test No.	No. of tested sporangiophores	PCR product ofType A	Type C	Type A+C
1	8	0	6	2
2	8	0	2	6
3	16	0	15	1
n = 3	32	0 (0%)	23 (72%)	9 (28%)

**Table 5:** PCR of sporangia. Amplification products detected in single sporangia collected from infected plants after dual inoculation with strain A+B (n = 3 inoculation experiments, repeated tests from the same inoculation are marked with a or b) and A+C (n = 1).

Test No.	No. of tested sporangia	PCR product ofType A	Type B or C	Type A+B /C	without PCR product
1	16	3	1 C	2	10
2a	16	3	3 B	3	7
2b	24	2	3 B	3	16
3a	24	0	7 B	2	15
3b	24	3	4 B	2	15
4	24	0	7 B	3	14
n = 4	128	11 (9%)	25 (20%)	15 (12%)	77 (60%)

**Table 6:** PCR of zoospores. Amplification products detected in single zoospores individually collected from sporangia of infected plants after dual inoculation with strain A+B (n = 5 inoculation experiments).

Test No.	No. of tested zoospores	PCR product ofType A	Type B	Type A+B	without PCR product
1	48	1	14	1	32
2	48	0	7	1	40
3	24	9	0	1	14
4	48	2	14	3	29
5	88	13	6	1	68
n = 5	256	25 (10%)	41 (16%)	7 (3%)	183 (71%)

**Fig 2:**
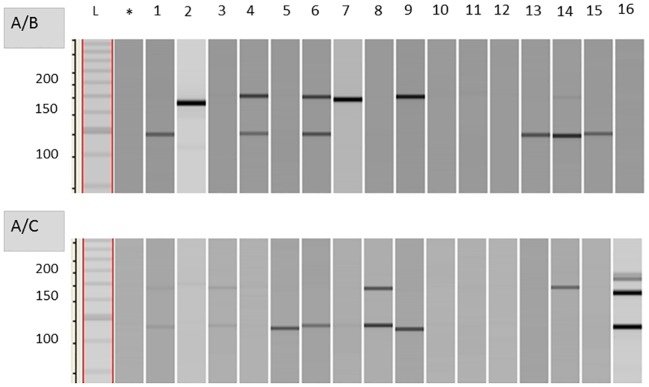
Amplification products of single sporangia. Single sporangia (lane 1–16) were collected from infected plants of the dual inoculation experiments with strain A+B (upper part) and A+C (lower part) and PCR products analysed by capillary electrophoresis. A/B: Lanes 1, 13, 14 show the 113 bp product characteristic for strain A; lanes 2, 7, 8 show the 165 bp product of strain B; lanes 4, 6, 14 show products of both progenitors in different amounts; lanes 3, 10, 11, 12, 16 gave no products. *, negative control with water; L, kb ladder; in the second experiment 7 lanes showed the band of either strain A (5, 6, 7, 9) or C (2, 4, 14), while 3 lanes (1, 3, 8) displayed a combination of both progenitors and 5 lanes (10, 11, 12, 15) failed to give products.

In PCR experiments with single sporangia, 60% of the samples failed to amplify the target sequences in detectable amounts (Table 5). However, 29% of the samples showed the PCR product of either of the parents and 12% contained both bands. The intensity of the two parental bands varied to each other (Fig 2) thus indicating that sporangia may contain nuclei of strain A and B (or C) in different proportions.

Single zoospores were tested only for the dual inoculation experiments of strain A + B. From 256 samples, 29% gave detectable PCR products of which 16% and 10% showed only one band of either strain A or B, but 3% revealed both bands in more or less equal amounts (Table 6). This indicates the presence of both parental templates in individual zoospores although these are generally assumed to be monokaryotic. Fluorescence microscopy after DNA staining with Hoechst 33342 or DAPI dyes did not show zoospores with more than one nucleus from our experiments (data not shown).

**Fig 3:**
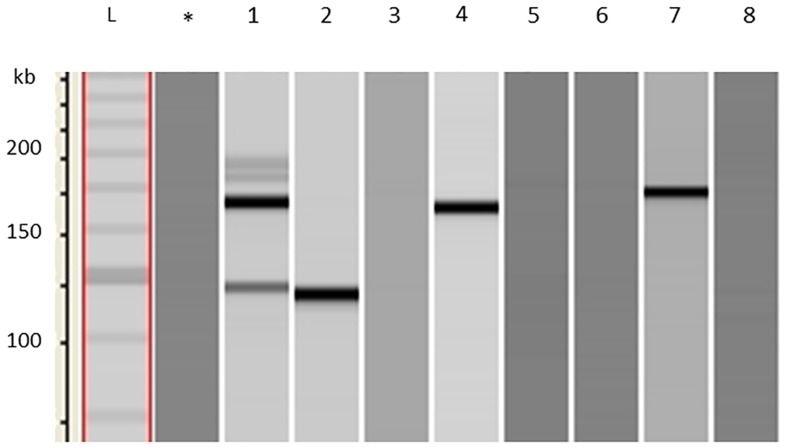
Amplification products of single zoospores. Zoospores (lane 1–8) were collected from a single sporangium of infected plants of the dual inoculation experiments with strain A+B and analysed by capillary electrophoresis. Lane 2 shows the 113 bp product characteristic for strain A; lanes 4 and 7, contain the 165 bp product of strain B; lane 1 displays both products; lanes 3, 5, 6 and 8 gave no amplicons. *, negative control with water; L, kb ladder.

When comparing zoospores released from the same sporangium, all three possible amplification patterns could be obtained (Fig 3). From eight collected sister zoospores, one gave the amplicon of strain A, two of strain B, 1 of a recombinant A+B and four failed to provide detectable amounts of PCR product. This result shows that sporangia may harbour at the same time individual zoospores of the genotype of each parental strain as well as zoospores of recombinant genotype (for illustration see Fig 1).

## Discussion

Previous studies involving whole seedling inoculation of sunflower simultaneously with two phenotypically different strains of *P*. *halstedii* suggested that asexual recombination in the course of the infection process is likely to account for inherited recombinant characters in the mitotically derived sporangial generations of the pathogen [14]. Moreover, ISSR fingerprints revealed genetic differences between the offspring generations and the parents, thus indicating that true genetic exchange and not just sledge effects between the used parental strains allowed infection. However, it remained unclear from these experiments, in which phase of the infection process such an exchange could occur, which pathogenic structures may be involved and whether or not the acquired infection characteristics are due to heterokaryon situation or true recombination.

The data presented here lent further support to the possibility of genetic exchange without gametangiogamy in *P*. *halstedii*. In addition, this process was shown not to be a rare event, but occured rather regularly and between different parental genotype combinations. Considering a previous report [24] according to which host independent stages of the pathogen (zoospore release, zoospores, germinating cystospores) are much less affected by metalaxyl than host dependent stages (e.g. young mycelia) it appears most likely that the genetic exchange between the tolerant strain A and the sensitive strains B or C takes place prior to establishing the mycelial stage of infection. This also has to be favored with respect to the selected pathogenicity of the used strains, which in contrast to the previous experimental design [14], impeded simultaneous development of both mycelia and the subsequent transfer of genetic material via anastomosis (Fig 1D). Although anastomosis have been shown to occur between hyphae of *P*. *halstedii in planta* [17], the hyphal fusion presumably would have to take place at the stage of germinating sporocysts prior to penetrating the host in the current experiment. In contrast to other filamentous fungi, where fusion of germ tubes has been documented [25–26], no such events have so far been reported from ooymcetes, and we have not found fused germ tubes in microscopic studies of host independent stages of *P*. *halstedii* (data not shown). Therefore, it appears more likely that the physical contact between the progenitors takes place in the zoospore phase, where cellular fusion is not impeded by the cell wall. Zoospore fusion has been postulated as a natural mode of asexual hybridization in *Phytophthora infestans* [15, 27] that produced interspecific hybrids via chemical-induced zoospore fusion. Despite intensive microscopic search (data not shown), we have not yet observed fusion stages when we mixed zoospores of strain A and B, but this could be due to experimental difficulties. Zoospores are rather delicate and easily burst before encystment if environmental conditions are unsuitable or stressful (e.g. osmolarity, temperature, ion concentration). Hence, it can also not be ruled out, that uptake in zoospores of external nuclei from bursted zoospores may lead to heterokaryon situation (Fig 1A) as it has been shown for *Phytophthora* [16].

The PCR-based identification of amplicons specific for both parental strains (A and B or A and C) in asexually produced sporangia from recombinant infections with *P*. *halstedii* clearly underlines that genetic exchange has taken place. The genotype of the fungicide resistant strain often dominated in our experiments (ca. 70% of sporangiophores, 45% of positive tested sporangia), which could be due to different vitality. The simultaneous presence of sporangia with either of the parental amplicons or in combination favours their origin from heterokaryotic hyphae. This would require that more than one nucleus enters the sporangium during the budding phase and that the zoospores of a sporangium from heterokaryotic hyphae could be of different genotype. This assumption is supported by microscopic studies [28], which indicated that the high number of zoospores in sporangia of *P*. *halstedii* (normally ranging between 25–30; [22]) resulted from immigration of several nuclei in the early budding stage before a callose septum separated the sporangium from the sterigmum. Subsequent cell division leads to the final number of zoospores. The identification of zoospores representing the genotype of strain A or B released from the same sporangium confirms the assumption of independent immigration of more than one nucleus in the budding phase.

In contrast to sporangia, a heterokaryon situation cannot be assumed for the PCR results of individual zoospores that showed amplification products of parents. Despite intensive search with fluorescence microscopy and staining with DAPI (data not shown), we have not identified any zoospores with more than one nucleus. This supports the assumption that true recombinant nuclei derived from dual inoculation assays. Further experiments will be necessary to test this hypthesis and to investigate the dimension of recombinant genetic exchange.

## Conclusions

Dual inoculation of sunflower with strains of *P*. *halstedii* complementary in pathogenicity conquered resistance barriers, which could not be overcome by either strain alone. The experimental design favours the assumption that parasexual events in the host-independent stage of infection enabled genetic exchange required to break the infection barriers. Presumably initiated by heterokaryolgy, PCR analysis in mononuclear zoospores indicated that true recombination could have taken place and could be inherited via single zoospores. This mode of genetic exchange appears be an important factor to explain the high genetic diversity found in sunflower downy mildew, a pathogen that usually relays on homothallic sexual reproduction. The results also imply that this type of recombination may be essential for host jumps in obligate biotrophic oomyctes [29] and could be an early key event in speciation.

## Supporting Information

S1 Fig PCR-products (inverted gel document) of strain A-C with primer LR6-Om (5‘-CGCCAGACGAGCTTACC). From lane A and B/C, the polymorphic bands at 930 bp (left arrow) and 980 bp (right arrow) were cloned and sequenced. Sequences of B and C were identical and differed from A in a 30 bp insertion.
(TIF)

S2 Fig Isolation of single zoospores from agar using microcapillary techique. (TIF)

S3 Fig Amplification with primer pair INBL_WG_F1/R1 of the sequence parts specific for strain A (113 bp product) and strain B/C (165 bp product) analysed by capillary electrophoresis. Lanes 2–6, products obtained from single sporangia of the strains A-C and mixtures of 1A/1B and 1A/1C sporangia; lanes 9–12, products obtained from 10, 5, 2, 1 zoospores of strain A; lanes 14–16, products obtained from 9, 2, 1 zoospores of strain B; *, negative control with water; L, kb ladder; PB, primer band; UM/LM, upper and lower marker.
(TIF)

S1 Table Primer sequences used for DNA fingerprint with strain A-C. (PDF)

S2 Table LR6 OM Sequence of BL-A4z (982 bp) and In-B5z (930 bp). Annealing sites for INBL_WG_F1/R1 in bold; 52 nt insert sequence of BL-A4z printed in lower case.
(PDF)
