# Supplementary figures and images for: Asexual Recombinants of Plasmopara halstedii Pathotypes from Dual Infection of Sunflower
Source: PLoS One. 2016 Dec 1;11(12):e0167015. doi: 10.1371/journal.pone.0167015 (PMC5132302; doi:10.1371/journal.pone.0167015)

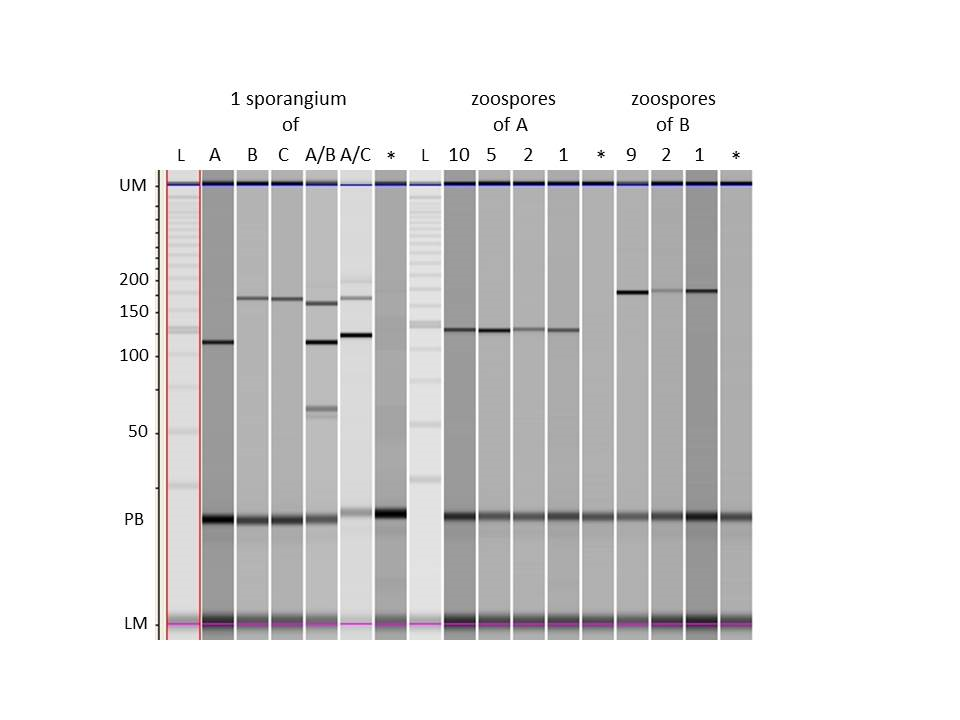

Supplement: S1 Fig — From lane A and B/C, the polymorphic bands at 930 bp (left arrow) and 980 bp (right arrow) were cloned and sequenced. Sequences of B and C were identical and differed from A in a 30 bp insertion. (TIF) [file pone.0167015.s001.tif]

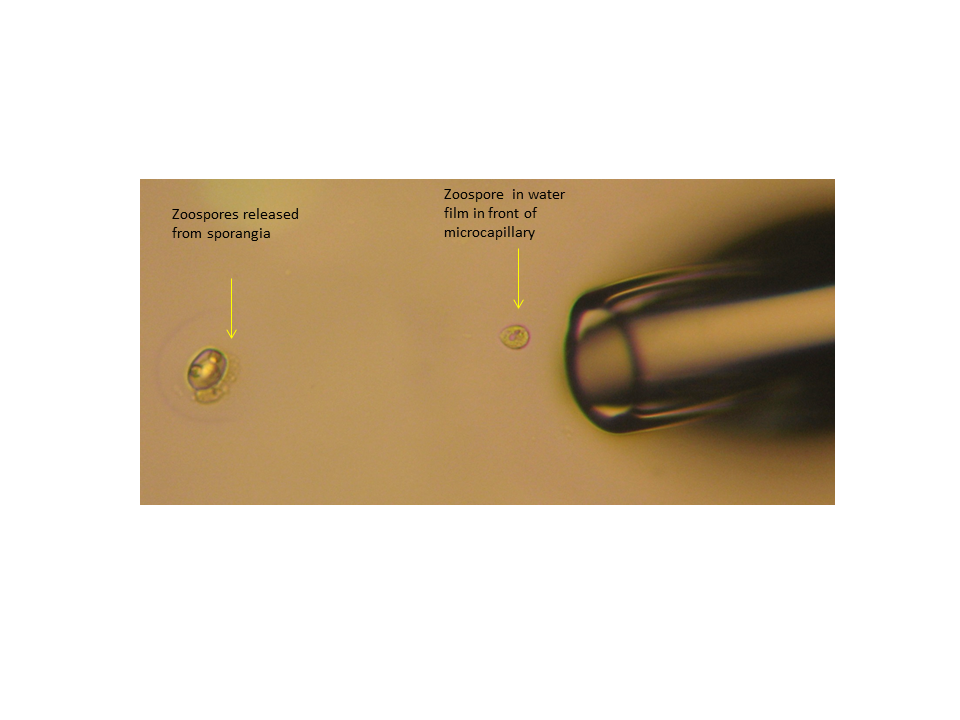

Supplement: S2 Fig — (TIF) [file pone.0167015.s002.tif]

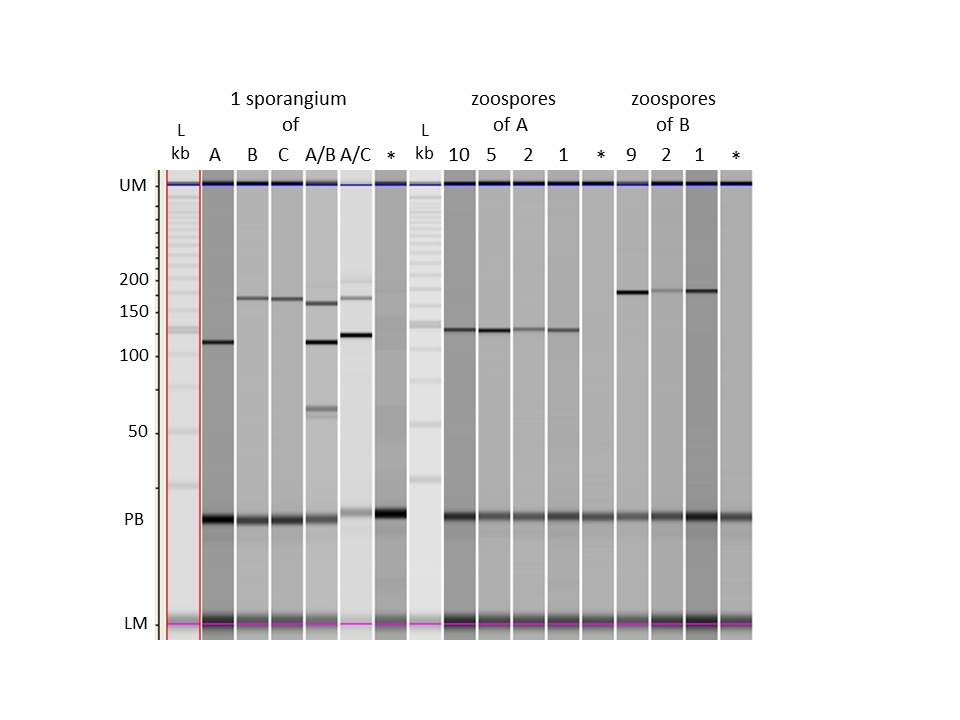

Supplement: S3 Fig — Lanes 2–6, products obtained from single sporangia of the strains A-C and mixtures of 1A/1B and 1A/1C sporangia; lanes 9–12, products obtained from 10, 5, 2, 1 zoospores of strain A; lanes 14–16, products obtained from 9, 2, 1 zoospores of strain B; *, negative control with water; L, kb ladder; PB, primer band; UM/LM, upper and lower marker. (TIF) [file pone.0167015.s003.tif]
