# Supplementary material for: Asexual Recombinants of Plasmopara halstedii Pathotypes from Dual Infection of Sunflower
Source: PLoS One. 2016 Dec 1;11(12):e0167015. doi: 10.1371/journal.pone.0167015 (PMC5132302; doi:10.1371/journal.pone.0167015)
Supplement: S1 Table — (PDF) [file pone.0167015.s004.pdf]

Supplementary 1 Table: Primer sequences used for DNA fingerprint with strain A-C.

5.8S_R	TTACGTATCGCATTTCGCAG
Peter_Rubisco_down	CCAAACGTGAATACCCCCCGAAGC
Peter_Rubisco_up	GCTCTACGTCTGGAAGATTTGCGA
Cox2 Hud R	CCATGATTAATACCACAAATTTCACTAC
Cox2 Hud F	GGCAAATGGGTTTTCAAGATCC
ITS4	TCCTCCGCTTATTGATATGC
LROR	ACCCGCTGAACTTAAGC
DC6 F	GACTGGATCCGAACCCTCTATCTCAAGCCG
tnrL_R	GGGGATAGAGGGACTTGAAC
LRO	GCTTAAGTTCAGCGGGT
LROR	GCTTAAGTTCAGCGGGT
LR6-Om	CGCCAGACGAGCTTACC
