# Supplementary material for: Asexual Recombinants of Plasmopara halstedii Pathotypes from Dual Infection of Sunflower
Source: PLoS One. 2016 Dec 1;11(12):e0167015. doi: 10.1371/journal.pone.0167015 (PMC5132302; doi:10.1371/journal.pone.0167015)
Supplement: S2 Table — Annealing sites for INBL_WG_F1/R1 in bold; 52 nt insert sequence of BL-A4z printed in lower case. (PDF) [file pone.0167015.s005.pdf]

Supplementary 2 Table: LR6 OM Sequence of BL-A4z (982 bp) and In-B5z (930 bp). Annealing sites for INBL_WG_F1/R1 in bold; 52 nt insert sequence of BL-A4z printed in lower case.

CCTGATAACCCTGATGGGCTTTGAAGGCGTGGAGCACCTTGTTGCGCATGAGCCCGTGGCCATAAACACGCGCTTAGAGG

CTTTTTCAAAAGCGGTAT**GAAGGGTCAGTGTCCACGCAAAAT**CGACCGACGAACCctttacggggaaatatgagtgccag

taggccgccgttcgccgacttggatttGAATGGGTTTTTTGTTCCTCGTGGAGGTCGAGATCAAACCTTTACAGGTCAG**G**

ATGTTTGGCTGTTAATTTCGACGTTCCGAAAGTTGTGATTTTAGAAAAAAAAATCTCATATCAGGAGTGTCGTGCATTTT

GTTTGACGCGCGACCGAACAAAAAGAATACAGAACGCGATGGCATTAAAGATGCCAATATGTGCATTCTTTTCGTACTTT

GATACGATCTCAACGTGCCACCAACATAGTGTGATCGTCGCGGCTAGCGGTTCTTACATTTTTGAGGCGAACACCAAGTT

GCTCTCTCATGACAGCTATTACACCAAAGCTTTGAATCGTGACCCAACTGTTTTGCCATCACTACAATACATACAACAGA

GTCCTTCACCCAGAAAGAGGTATTGCCTACTCTCTGTCTTTATTATGCCTGTATGCTCGTATCCTACGGAGATAAGTGCT

GTGACACCTCGCCCAAATATCACGACTAACTCGTCACCAATTTGCTGACCTGGTCTAAATTTCTTTATACTTTTGTCCGA

CAGCAAGCGCCCGGTCTAAATTTTCTATTTATTATTTTGCCCGTCAGCAAGCGTCGCAAAAACATTTGCCAACACGATCT

AGTGTTTGGATGTAAATTTATCTTGGGTTTCTAGCCGCGACATGTTCAAGTTTCTATACGACTATACGTGCAGGCTTTCT

ACTGAAACGGAGCGAAGTGGGCGCTTGCTTAACCACAGGTCGTAGCTCTTAGGAACCTCTCCTGCGCGTGGAGCAGCATT

GGTCGGGTAAGCTCGTCTGGCG
